# Palladium-Catalyzed Synthesis of Aldehydes from Aryl Iodides and Formic acid with Propylphosphonic Anhydride as the Activator

**DOI:** 10.1038/s41598-018-26850-2

**Published:** 2018-05-30

**Authors:** Xiao-Feng Wu

**Affiliations:** 1 Leibniz-Institut für Katalyse e. V. an der Universität Rostock, Albert-Einstein-Straβe 29a, 18059 Rostock, Germany; 2 Department of Chemistry, Zhejiang Sci-Tech University, Xiasha Campus, Hangzhou, 310018 People’s Republic of China

## Abstract

An interesting palladium-catalyzed carbonylative procedure for the synthesis of aromatic aldehydes from aryl iodides has been developed. By using propylphosphonic anhydride as the activator for formic acid, moderate to good yields of the corresponding aldehydes were produced with formic acid as the carbonyl and hydrogen donors. Interestingly, neither additional phosphine ligand nor inert gas protection is needed here.

## Introduction

Aromatic aldehyde is an analogue of impotent chemicals with various usages in countless areas including pharmaceuticals, advanced materials and so on^1^. Furthermore, aromatic aldehydes are applied in fine chemicals synthesis as well^2^. Based on their importance, many synthetic procedures have been developed by organic chemists during the past years^3^. Among them, name reactions including Duff reaction^4^, Casiraghi reaction^5^, Gattermann-Koch reaction, Reimer-Tiemann reaction and so on^6^, have been established. Additionally, the oxidation of benzylic alcohols^7^ and methylarenes^8^ has been developed for aromatic aldehydes synthesis as well. Nevertheless, drawbacks such as relatively strict reaction conditions and low reaction efficiency limited the value of those procedures.

On the other hand, palladium-catalyzed carbonylation reactions represent a straightforward choice for carbonyl-containing compounds construction, including esters, amides, acids, and etc^9^. In the known carbonylative transformations, reductive carbonylation of aryl halides can provide aromatic and vinyl aldehydes in an efficient and straightforward manner^10^. In 1974, Heck and co-workers reported their pioneering studies on this topic^11^, since then significant improvements have been achieved during the past decades. For example, in 2006, Beller’s group reported an interesting and efficient palladium-catalyzed reductive carbonylation of (hetero)aryl bromides under the pressure of syngas (CO:H_2_ = 1:1)^12^. Even though carbon monoxide is one of the cheapest C1 source and holds non-replaceable position in large scale applications, the high toxicity, flammable and autoclave usage for its handling still limited the applications of CO gas based carbonylation in small scales. Under all those backgrounds, many new CO surrogates have been developed and applied in carbonylation reactions. The research group of Manabe prepared *N*-formylsaccharin and explored it as CO source for palladium-catalyzed reductive carbonylation of aryl bromides together with silane as the reductant^13^. The reaction efficiency is promising. Additionally, other CO sources including 9-methylfluorene-9-carbonyl chloride^14^, CO_2_^15^, paraformaldehyde^16^, and acetic formic anhydride^17^ have been explored in this topic by different groups as well. However, the requirement of expensive reducing reagents such as silanes and metal hydrides are one of the drawbacks. More recently, we established a novel palladium-catalyzed reductive carbonylation procedure for the synthesis of aromatic aldehydes from aryl iodides^18,19^. By using acetic anhydride as the activator, formic acid can be used both as the CO and hydride sources. High yields of the corresponding aldehydes were produced. During our studies on carbonylation reactions, propylphosphonic anhydride comes into our view^20–24^. Propylphosphonic anhydride has been applied in carboxylic acids activation, and we believe it can activate formic acid to release CO as well. Potentially, the produced by-product, propylphosphonic acid, can acting as ligand to stabilize the active palladium center and make the addition of additional phosphine ligand not necessary. With this original idea in mind, we started the studies of applying propylphosphonic anhydride in the reductive carbonylation of aryl iodides.

## Results

Initially, we chose iodobenzene as the model substrate to establish this idea (Table 1). Using the combination of Pd(OAc)_2_ and PPh_3_ as the catalytic system, with formic acid as the source of formyl group and NEt_3_ as the base in DMF at 100 °C for 5 hours, 10% of benzaldehyde was formed with the total conversion of iodobenzene (Table 1, entry 1). Interestingly, the reaction was totally inhibited when pyridine was used as the base (Table 1, entry 2). Then the reaction was tested without phosphine ligand, and even better yield of benzaldehyde was formed (Table 1, entry 3). Subsequently, the amounts of formic acid and propylphosphonic anhydride were tested, and 80% of benzaldehyde can be produced with higher loading of propylphosphonic anhydride (Table 1, entry 5). The conversion of iodobenzene decreased when the reaction was carried out at lower temperature (Table 1, entry 6). To our surprise, the same arrange yield of benzaldehyde can be formed with 2.5 mmol of NEt_3_ (Table 1, entry 7). This phenomenon implies that propylphosphonic acid as the produced by-product not necessarily to be neutralized. However, no reaction occurred in the absence of base (Table 1, entry 8). Then several other solvents were tested, but no improved yield can be obtained (Table 1, entries 9–13).

**Table 1:** Optimization of Reaction Conditions^a^.

Entry	Ligand	Base	Formic acid	T3P	Solvent	Temp.	Conversion^b^	Yield^b^
1	PPh_3_ (3 mol%)	Et_3_N (5 mmol)	4.5 mmol	0.5 mmol	DMF	100 °C	100%	10%
2	PPh_3_ (3 mol%)	Pyrdine (5 mmol)	4.5 mmol	0.5 mmol	DMF	100 °C	0%	0%
3	/	Et_3_N (5 mmol)	4.5 mmol	0.5 mmol	DMF	100 °C	98%	22%
4	/	Et_3_N (5 mmol)	6 mmol	0.5 mmol	DMF	100 °C	98%	26%
5	/	Et_3_N (5 mmol)	4.5 mmol	0.8 mmol	DMF	100 °C	100%	80%
6	/	Et_3_N (5 mmol)	4.5 mmol	0.8 mmol	DMF	80 °C	90%	60%
**7**	**/**	**Et**_**3**_ **N (2.5 mmol)**	**4.5 mmol**	**0.8 mmol**	**DMF**	**100** °C	**100%**	**82%**
8	/	/	4.5 mmol	0.8 mmol	DMF	100 °C	0%	0%
9	/	Et_3_N (2.5 mmol)	4.5 mmol	0.8 mmol	toluene	100 °C	100%	67%
10	/	Et_3_N (2.5 mmol)	4.5 mmol	0.8 mmol	H_2_O	100 °C	10%	2%
11	/	Et_3_N (2.5 mmol)	4.5 mmol	0.8 mmol	MeCN	100 °C	100%	55%
12	/	Et_3_N (2.5 mmol)	4.5 mmol	0.8 mmol	tBuOH	100 °C	100%	48%
13	/	Et_3_N (2.5 mmol)	4.5 mmol	0.8 mmol	DMSO	100 °C	100%	39%

^a^Reaction conditions: air, iodobenzene (1 mmol), Pd(OAc)_2_ (1.5 mol%), solvent (2 mL), 100 °C for 5 h. ^b^Yield and conversion were determined by GC with hexadecane as internal standard. T3P = propylphosphonic anhydride.

**Figure 1:**
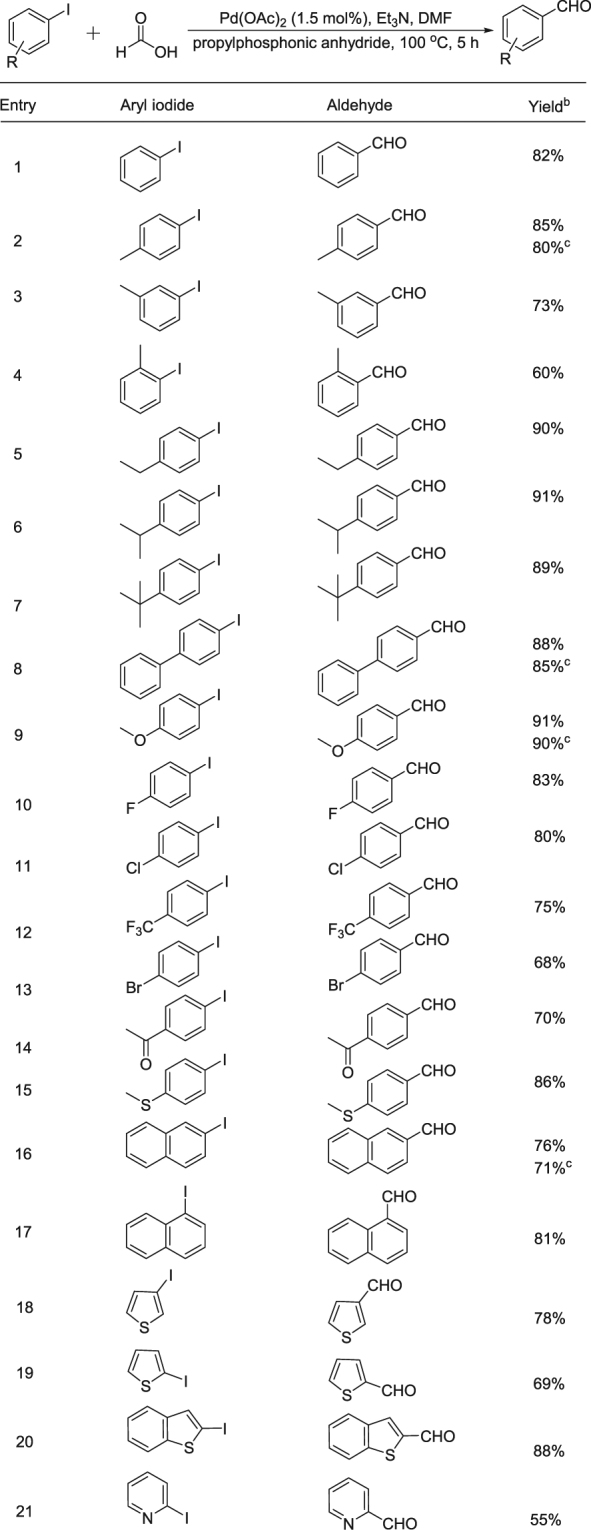
Synthesis of aldehydes from aryl iodides^a^. ^a^Reaction conditions: under air, aryl iodide (1 mmol), Pd(OAc)_2_ (1.5 mol%), formic acid (4.5 mmol), propylphosphonic anhydride (0.8 mmol), Et_3_N (2.5 mmol), DMF (2 mL), 100 °C for 5 h, isolated yields (see supporting information). ^b^Yield and conversion were determined by GC with hexadecane as internal standard. ^c^Isolated yields.

After established the optimum catalytic system, we started the scope and limitation testing. As shown in Fig. 1, moderate to good yields of the corresponding aldehydes can be produced in general. Both electron-donating and electron-withdrawing substituents on the aromatic iodides can be well tolerated. 2-Iodonaphthalene and 1-iodonaphthalene are suitable substrates as well, good yields of the desired aldehydes were produced (Fig. 1, entries 16–17). Heterocyclic substrates can be applied and smoothly transformed as well, moderate to good yields of the corresponding products can be detected (Fig. 1, entries 18–21).

## Discussion

**Figure 2:**
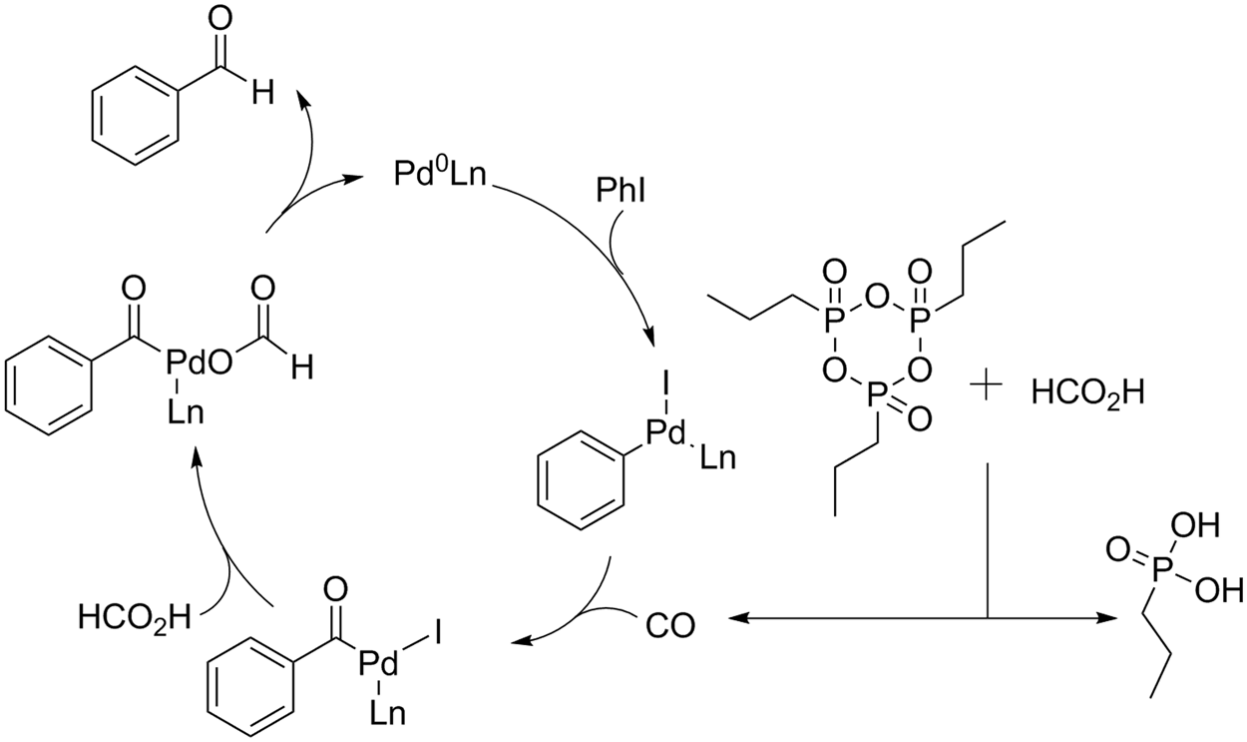
Proposed reaction pathway.

A plausible reaction mechanism is proposed and shown in Fig. 2 as well. Initially, oxidative addition of aryl iodide to Pd(0) species generates an arylpalladium complex. Then, carbon monoxide, prepared *in-situ* from formic acid, can be inserted into the arylpalladium complex to give an aroylpalladium species. Finally, ligand exchange of the aroylpalladium complex with another molecular of formic acid leads to an acylpalladium formic acid complex, which undergoes decarboxylation and reductive elimination to give the expected aldehyde product and regenerate the Pd(0) species.

In summary, an attractive palladium-catalyzed carbonylative procedure for transforming aryl iodides into the corresponding aldehydes has been developed. By using propylphosphonic anhydride as the activator for formic acid, moderate to good yields of the corresponding aldehydes can be formed with formic acid as the carbonyl and hydrogen donors. Interestingly, neither additional phosphine ligand nor inert gas protection is needed here.

## Methods

### General Procedure

Under air, Pd(OAc)_2_ (0.03 mmol, 1.5 mol%) was added to an oven-dried tube. Then aryl iodide (1 mmol), DMF (2 mL), HCO_2_H (4.5 mmol), NEt_3_ (2.5 mmol), and propylphosphonic anhydride (0.8 mmol; 50% in DMF) were added to the reaction tube via syringe. Subsequently, the tube was sealed and stirred at 100 °C for 5 h. Then the tube was cooling down to room temperature and 100 mg of hexadecane was added into the tube as internal standard. After properly mixed, a part of the mixture was subjected to GC analysis for determination of the yield and conversion.

## Electronic supplementary material

Supporting Information
