# Supplementary material for: Palladium-Catalyzed Synthesis of Aldehydes from Aryl Iodides and Formic acid with Propylphosphonic Anhydride as the Activator
Source: Sci Rep. 2018 May 30;8:8389. doi: 10.1038/s41598-018-26850-2 (PMC5976648; doi:10.1038/s41598-018-26850-2)

Supporting Information

Palladium-Catalyzed Synthesis of Aldehydes from Aryl Iodides and Formic acid with Propylphosphonic Anhydride as the Activator

Xiao-Feng Wu*

Leibniz-Institut für Katalyse e. V. an der Universität Rostock, Albert-Einstein-Straβe 29a, 18059 Rostock, Germany

E-mail: xiao-feng.wu@catalysis.de

4-Methylbenzaldehyde

Light yellow oil. ^1^H NMR (400 MHz, CDCl_3_) δ 10.0 (s, 1H), 7.85 (d, *J* = 7.9 Hz, 2H), 7.37 (d, *J* = 7.8 Hz, 2H), 2.48 (s, 3H). ^13^C NMR (101 MHz, CDCl_3_) δ 192.0, 145.5, 134.1, 129.8, 129.6, 117.2, 21.8.

**4-Methoxybenzaldehyde**

Light yellow oil. ^1^H NMR (400 MHz, CDCl_3_) δ 9.90 (s, 1H), 7.84 (d, *J* = 8.8 Hz, 2H), 7.02 (d, *J* = 8.7 Hz, 2H), 3.90 (s, 3H). ^13^C NMR (101 MHz, CDCl_3_) δ 191.0, 164.8, 132.1, 129.9, 114.5, 55.6.

**4-Biphenylcarboxaldehyde**

White solid. ^1^H NMR (400 MHz, CDCl_3_) δ 10.11 (s, 1H), 7.98 (d, *J* = 8.3 Hz, 2H), 7.80 (d, *J* = 8.2 Hz, 2H), 7.79 (d, *J* = 8.5 Hz, 2H), 7.54 (t, *J* = 7.6 Hz, 2H), 7.47 (t, *J* = 7.1 Hz, 1H). ^13^C NMR (101 MHz, CDCl_3_) δ 191.9, 147.2, 139.7, 135.2, 130.3, 129.0, 128.5, 127.7, 127.3.

**2-Naphthaldehyde**

Light yellow solid. ^1^H NMR (400 MHz, CDCl_3_) δ 10.20 (s, 1H), 8.37 (s, 1H), 7.93 – 8.05 (m, 4H), 7.61-7.71 (m, 2H). ^13^C NMR (101 MHz, CDCl_3_) δ 192.3, 136.5, 134.6, 134.1, 132.6, 129.5, 129.1, 128.1, 127.1, 122.7.


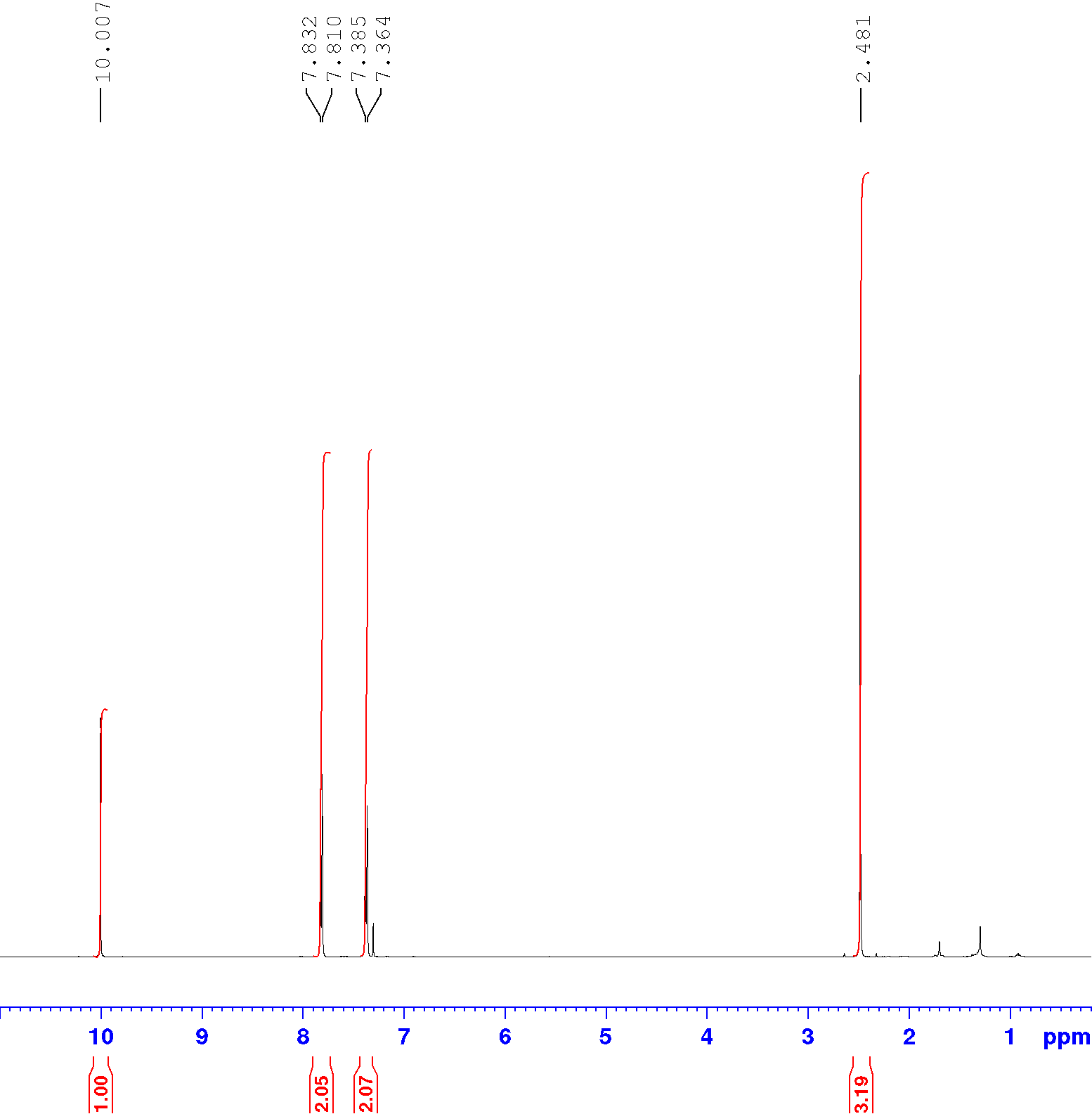


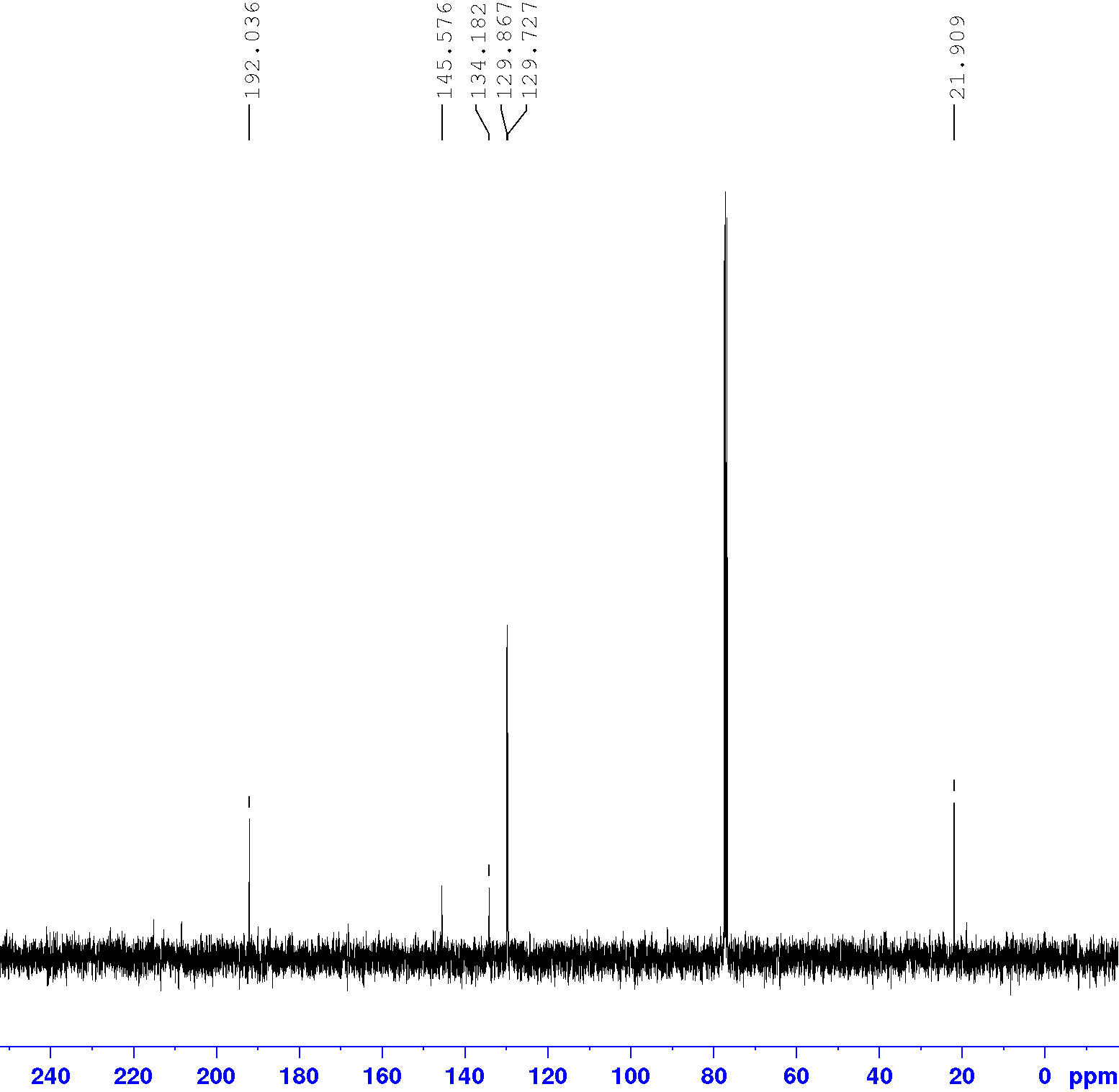


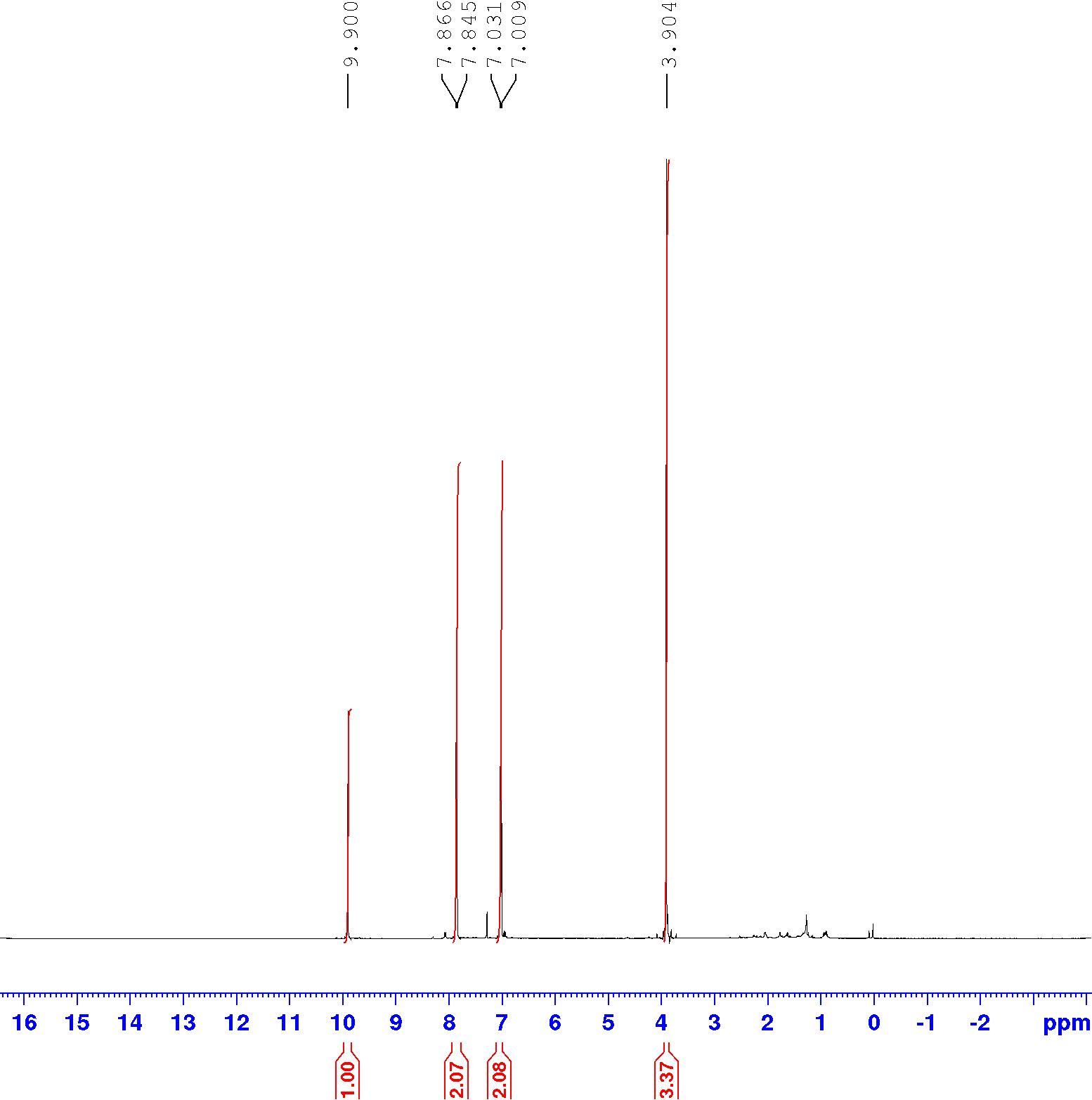


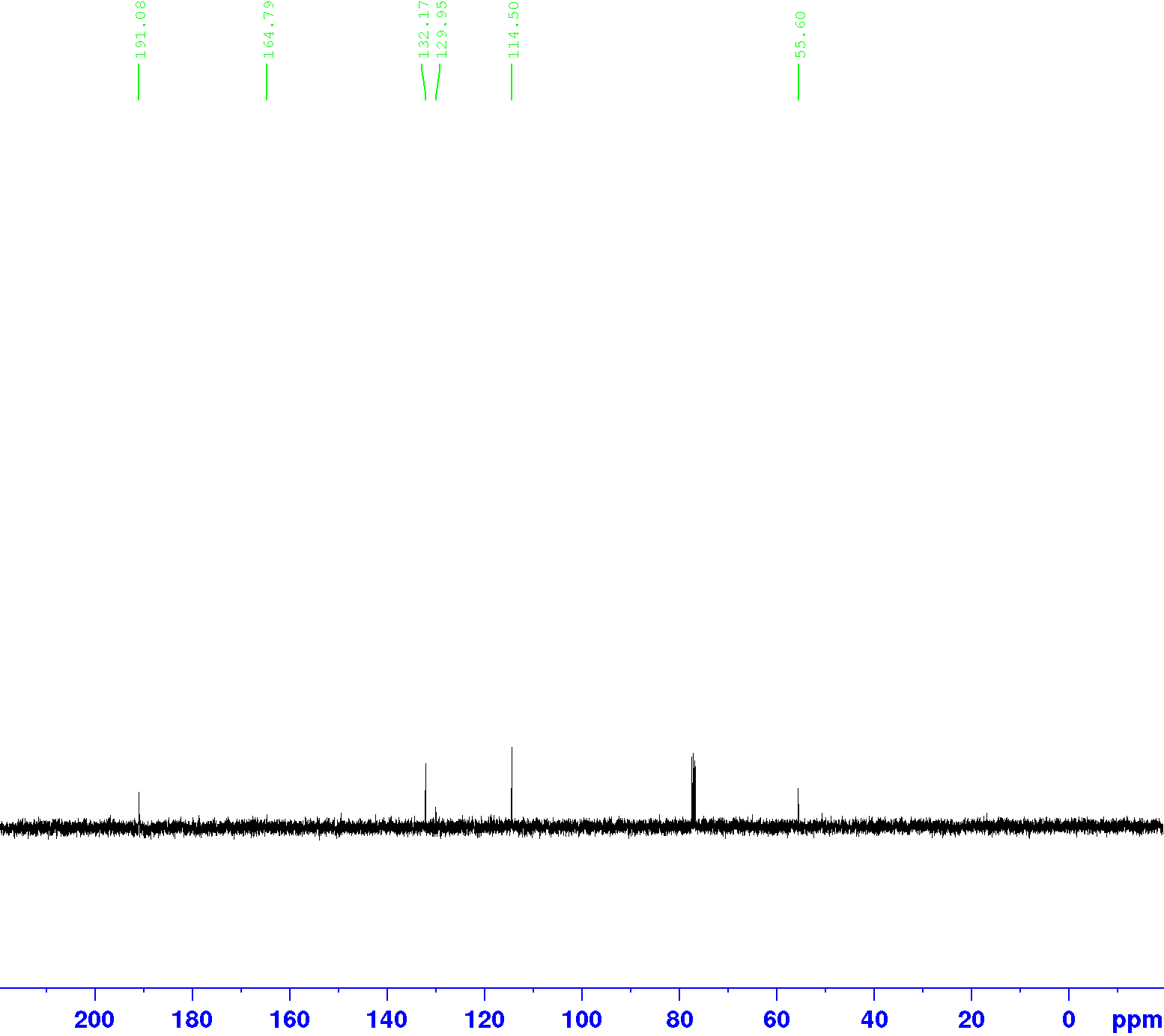


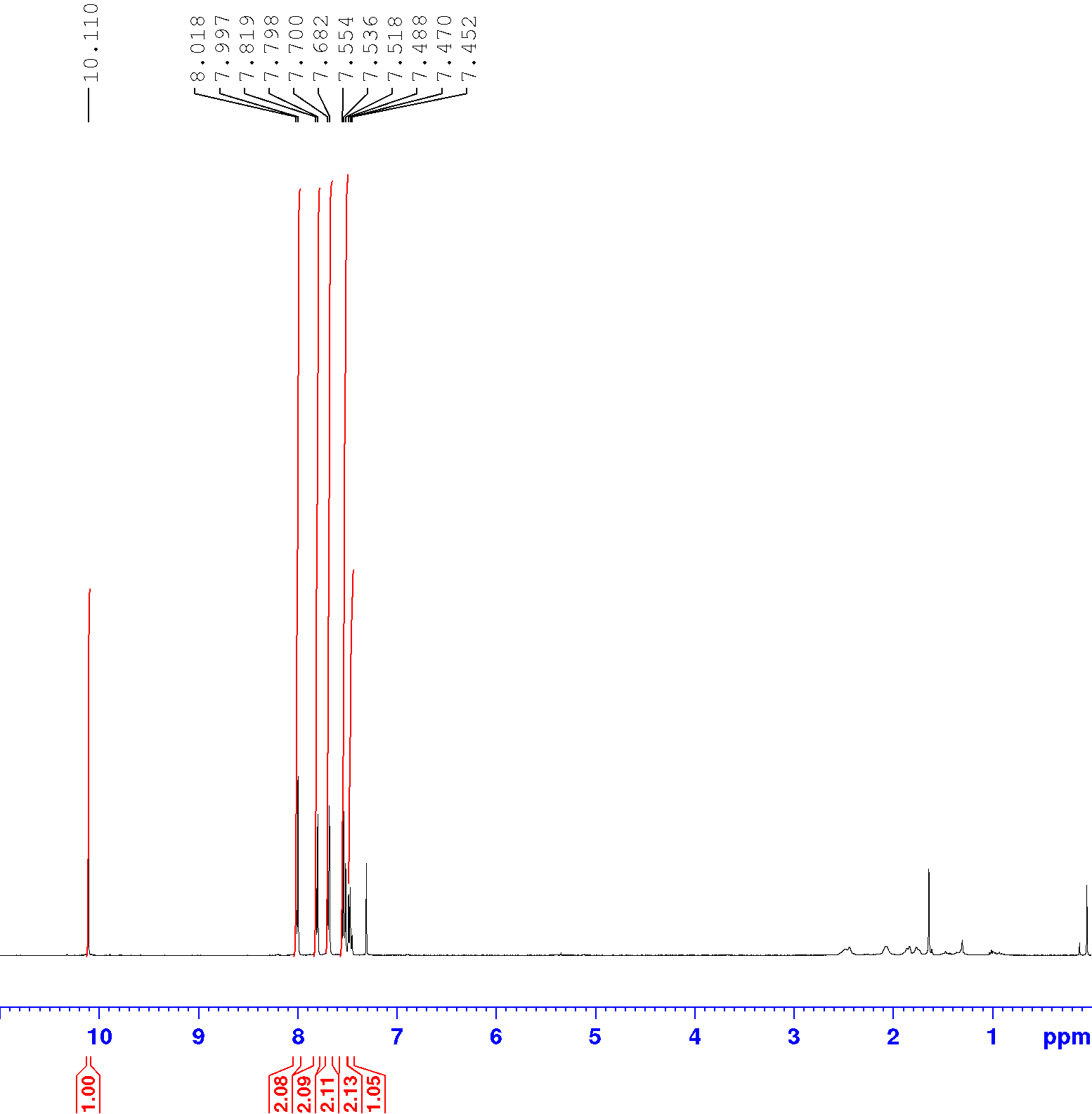


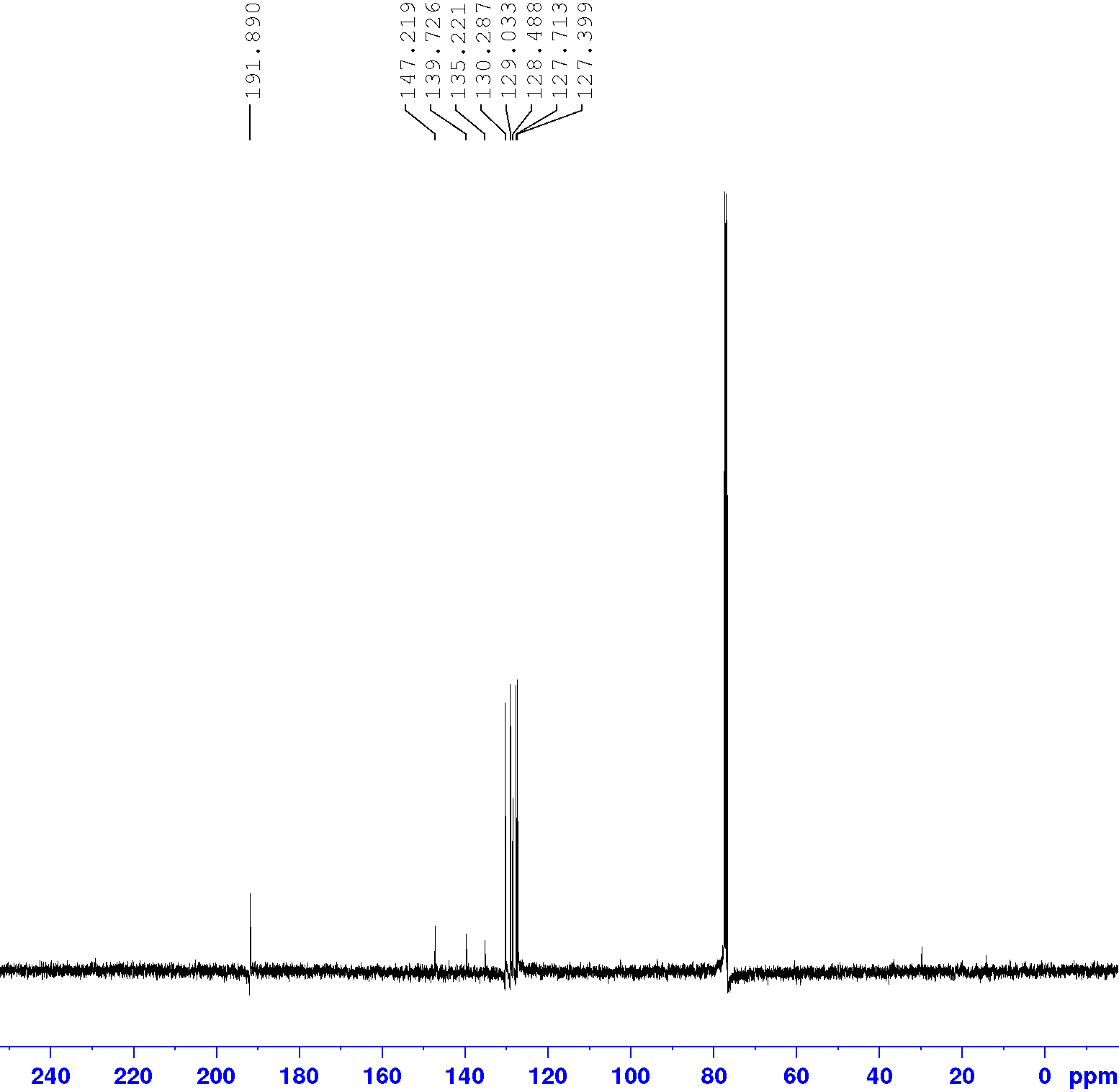


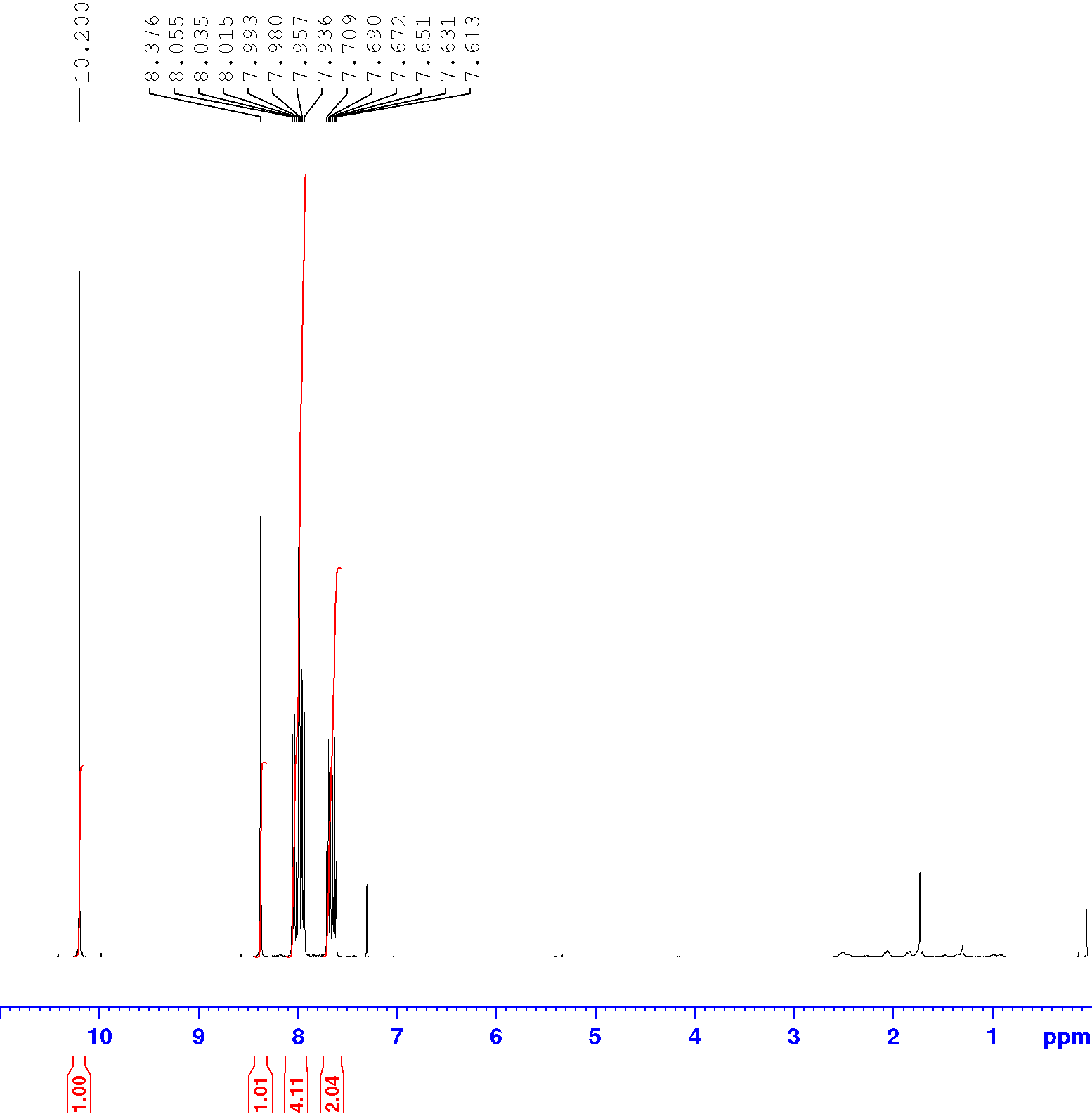


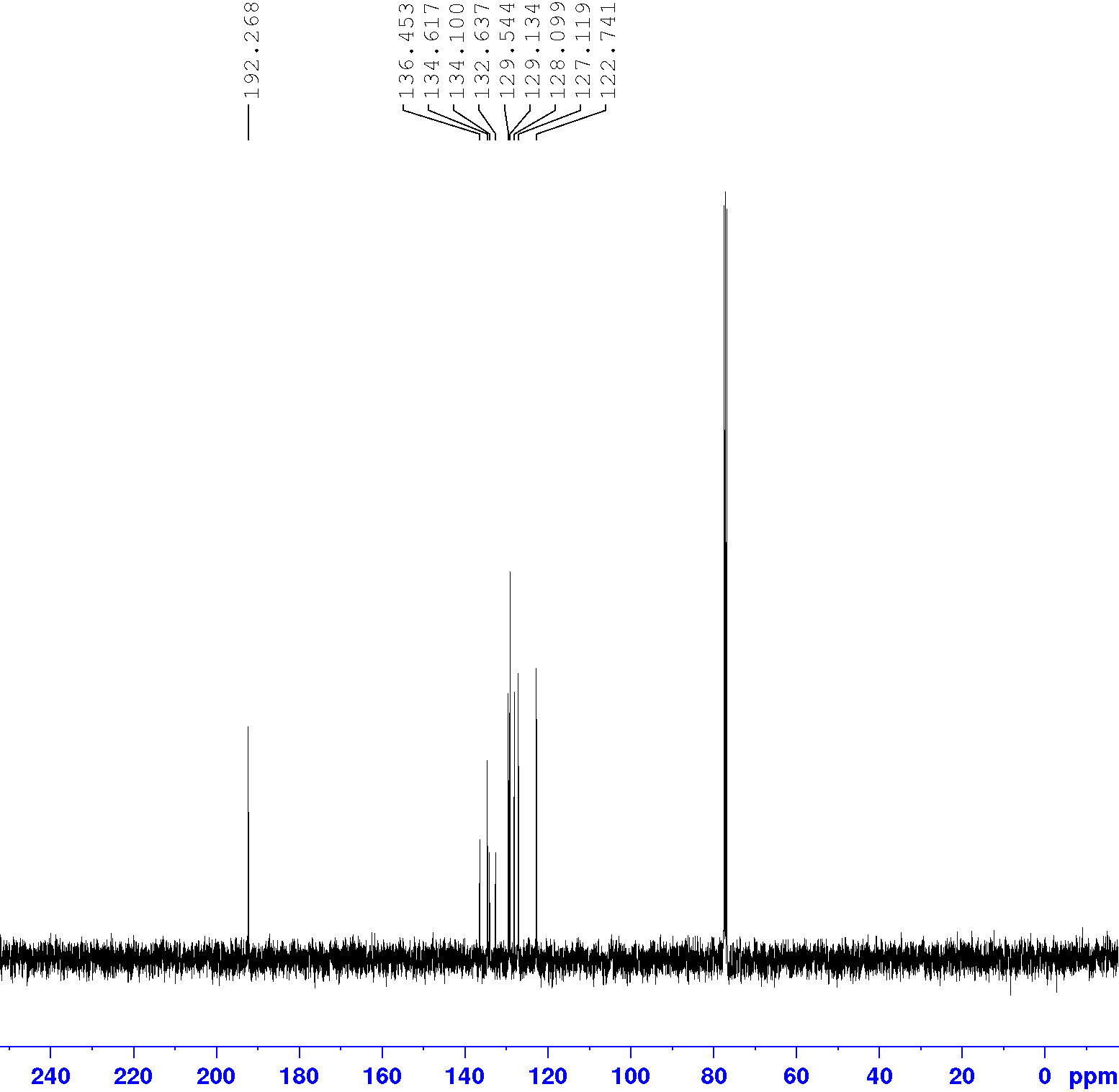

Supplement: Supplementary file 1 — Supporting Information [file 41598_2018_26850_MOESM1_ESM.docx]
